# The novel GLP-1/GIP dual receptor agonist DA5-CH is superior to tirzepatide and exendin-4 in the 6-OHDA Parkinson rat model

**DOI:** 10.3389/fendo.2026.1825379

**Published:** 2026-05-05

**Authors:** Zhang Lingyu, Feng Peng, Zhong Wanting, Jin Qianqian, Zijuan Zhang, Bo Bai, Guofang Xue, Christian Hölscher

**Affiliations:** 1 Experimental Animal Center of Shanxi Medical University, Shanxi Key Laboratory of Human Disease and Animal Models, Taiyuan, Shanxi, China; 2 Second Hospital, Neurology Department, Shanxi Medical University, Taiyuan, Shanxi, China; 3 School of Medical Science, Shanxi Medical University, Taiyuan, Shanxi, China; 4 Department of Forensic Pathology, Shanxi Medical University, Taiyuan, Shanxi, China; 5 School of Medical Sciences, Henan University of Chinese Medicine, Zhengzhou, Henan, China

**Keywords:** cytokines, GLP-1, growth factor, inflammation, insulin, mitochondria

## Abstract

**Introduction:**

Parkinson’s disease (PD) is a progressive neurodegenerative disorder for which there is no cure. Diabetes is one of the risk factors for developing PD. Tirzepatide is a novel long-acting glucagon-like peptide-1 (GLP-1) and glucose-dependent insulinotropic polypeptide (GIP) receptor agonist that is on the market as a treatment for diabetes. Importantly, two phase II trials in PD patients showed good effects with the GLP-1 receptor agonists Exendin-4 and Lixisenatide.

**Methods:**

We have developed a dual GLP-1/GIP receptor agonist (DA5-CH) that can cross the blood-brain barrier (BBB) at a higher rate than Tirzepatide. Here, we tested Exendin-4, Tirzepatide and DA5-CH in the 6-OHDA-lesion rat model of PD. The drug treatment was daily (10 nmol/kg, ip.) for 30 days.

**Results:**

DA5-CH was more effective than Tirzepatide or Exendin-4. In the substantia nigra, dopaminergic neurons were protected, with DA5-CH being most effective. Dopamine levels in the striatum were normalized by DA5-CH, while Exendin-4 was less effective, and Tirzepatide was ineffective. The inflammation response in the lesioned striatum was reduced by the drugs as shown in reduced IL-6 and TNF-α levels, with DA5-CH being more effective than Exendin-4, and Tirzepatide showing minimal effects. Furthermore, the α-synuclein levels in the substantia nigra were reduced by DA5-CH, superior to Exendin-4 and Tirzepatide.

**Discussion:**

Therefore, DA5-CH was more effective and may be a better therapeutic drug for neurodegenerative disorders such as PD compared to Tirzepatide or Exendin-4.

## Introduction

Parkinson’s disease (PD) is a chronic neurodegenerative disorder for which there are no disease-modifying treatments available. A key risk factor for developing PD is type 2 diabetes (T2DM) (1–4). Glucagon-like peptide 1 (GLP-1) is an incretin hormone that can improve T2DM. Several GLP-1 receptor agonists are on the market to treat T2DM. GLP-1 receptor agonists have shown protective effects in PD clinical trials (5–7) and Alzheimer’s disease (8). Another incretin hormone is glucose-dependent insulinotropic polypeptide (GIP), which has anti-diabetic properties also (9–12). Tirzepatide, a novel dual GLP-1/GIP receptor agonist, has been brought to the market to treat T2DM (13). GLP-1 receptor agonists have neuroprotective effects in animal models of PD (14, 15), and show effects in clinical trials that tested these drugs in patients with PD (5, 7, 16). GIP receptor agonists have shown protective effects in animal models of PD, too (17). We have developed novel dual GLP-1/GIP receptor agonists that can cross the blood-brain barrier (BBB) better than the drugs on the market that have been developed to treat T2DM (18–20). These dual agonists have previously shown good effects in animal models of PD (15). We have previously tested the dual agonist DA5-CH in the 6-OHDA rat model of PD and found that it shows neuroprotective effects that are superior to that of the GLP-1R agonist semaglutide (21). Motor activity was preserved and dopaminergic neurons protected more by DA5-CH treatment compared to semaglutide treatment. In addition, the level of α-synuclein was much reduced by DA5-CH compared to semaglutide. In the present study, we tested the novel dual GLP-1/GIP receptor agonist Tirzepatide in the same rat model and compared it to the effects of DA5-CH and the GLP-1R agonist Exendin-4. Tirzepatide is currently on the market as a treatment for diabetes (Mounjaro) and obesity (Zepbound) (13, 22).

## Material and methods

### Drugs, antibodies and reagents

Exendin-4 and Tirzepatide had been purchased from Macklin Reagent (China), and DA5-CH was synthesized by GL Biochem (China). Peptides were reconstituted in ultrapure water to a concentration of 1 mg/ml, and aliquots were prepared and stored at -20°C. The following antibodies were used in western blot assay or Immunohistochemistry (IHC): anti-tyrosine Hydroxylase antibody (1: 500; Boster, China), anti-α-Synuclein antibody (1: 500; Boster, China), anti-GFAP antibody (1: 200; Boster, China), anti-IBA-1 antibody (1: 200; Boster, China), anti-TNF-α antibody (1: 200; Boster, China), Anti-GAPDH (1: 2000; Boster, China), secondary antibodies (1: 5000; Boster, China) and a peroxidase-conjugated goat anti-rabbit secondary antibody (1: 200; Boster, China). The following reagents were used in Quantitative reverse transcription (qRT) PCR: SweScript All-in-One RT SuperMix for the qPCR Kit (Servicebio, China), gDNA Remover Kit (Servicebio, China), and 2×Universal Blue SYBR Green qPCR Master Mix Kit (Servicebio, China). Other reagents include 6-hydroxydopamine hydrobromide (6-OHDA, Sigma, USA), pargyline hydrochloride (MedChemExpress, USA), desipramine hydrochloride (MedChemExpress, USA), apomorphine (Absin, China), paraformaldehyde (PFA, Boster Biotechnology, China), Radio Immunoprecipitation Assay (RIPA) buffer (Beyotime, China), phenyl-methylsulfonyl fluoride (PMSF, Beyotime, China), BCA Protein Assay Kit (Boster, China), ECL- enhanced chemoluminescence Kit (Boster, China).

### Animals, stereotactic surgery, and drug administration

**Figure 1:**
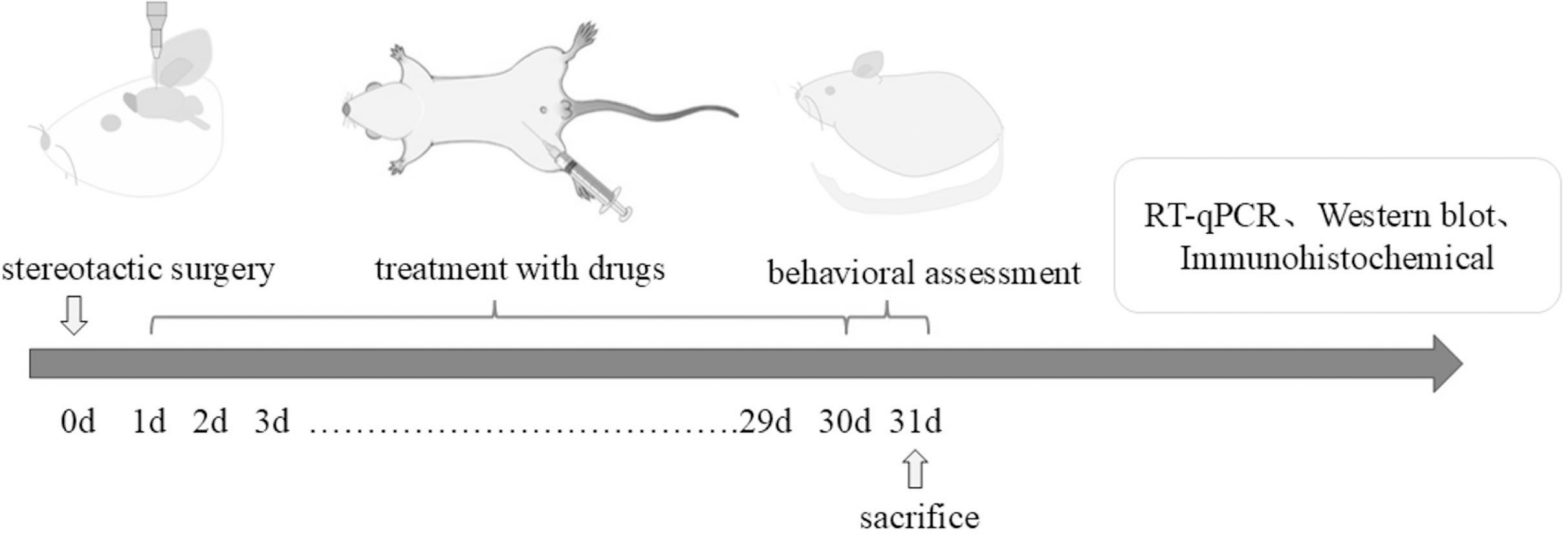
Study design.

All animal experiments were conducted strictly with the National Institutes of Health Guide for the Care and Use of Laboratory Animals. Moreover, the ethics board of the Henan Academy approved all experimental procedures. This study utilized 50 three-month-old adult male Sprague-Dawley (SD) rats weighing 220–250 g and sourced from the Experimental Animal Center of the university. All animals had ad libitum admission to water and a standard rat diet and were housed in the animal room under a precisely regulated 12-hour light/12-hour dark cycle. Environmental conditions were maintained at 23 °C ± 2 °C and a humidity level of 55% ± 10%. Thirty minutes before the stereotactic surgery, the rats were administered pargyline (5 mg/kg, a monoamine oxidase inhibitor) and desipramine (10 mg/kg, a noradrenaline uptake inhibitor). Following the induction of general anesthesia via intraperitoneal injection of ketamine (100 mg/kg body weight) and xylazine (10 mg/kg body weight), the rats were securely positioned in a standard stereotaxic apparatus (RWD, China). A single injection of 6-OHDA (5 μl, 2 mg/ml, containing 0.05% ascorbic acid) was carefully delivered into the right medial forebrain bundle (MFB) at the coordinates: A = −2.2 mm anterior to bregma, L = −1.5 mm lateral to bregma, and V = –8.0 mm ventral to the dura. After the injection, the syringe was left in place for 10 minutes to allow for proper diffusion before being slowly withdrawn. Subsequently, the rats were randomly assigned to one of five groups, with each group consisting of 10 rats. Group sizes were estimated according to previous studies that utilized these techniques. Animals were randomized across cages to avoid group effects. The groups were as follows: the sham + saline group, the 6-OHDA + saline group, the 6-OHDA + Exendin-4 group, the 6-OHDA + Tirzepatide group, and the 6-OHDA + DA5-CH group. For the groups in the 6-OHDA + drug category, the rats were intraperitoneally injected with 10 nmol/kg body weight of the respective drug once daily for 30 days (as seen in **Figure 1**). In contrast, the sham group received an equivalent volume of saline.

### Apomorphine rotation test

The test was conducted thirty days after the surgery, when the rats had achieved complete postsurgery recovery. Rats were given a subcutaneous injection of apomorphine (0.05 mg/kg), placed individually in a square open Beld (diameter: 75 cm), and tracked using a computer tracking system (EthoVision XT software, Noldus Information Technology, Netherlands) for 30 min. Completed contralateral rotations were recorded by two examiners that were blinded to animal status. Prior to the behavioral test, animals were habituated to the test room overnight.

### Open-field assessment

The open-field test measures spontaneous activity, which is reduced by impaired dopaminergic synaptic transmission. The apparatus was composed of a 75cm diameter square area surrounded by a 35cm high wall. The rat was placed in the center of the open field and its movements were observed for 10 min. The distance of travel and tracks were measured by a computer tracking system (EthoVision XT software, Noldus information technology, Netherlands). After each run, the open field was cleaned with a 70% ethanol solution to prevent the build-up of olfactory cues.

### Western blotting

Brain tissues were carefully homogenized and lysed in ice-cold RIPA lysis buffer supplemented with phenyl-methylsulfonyl fluoride (PMSF). The lysates were centrifuged at 12,000 revolutions per minute (rpm) for 20 minutes at 4°C. The protein concentration of the total lysates was accurately determined using the BCA Protein Assay Kit. Equal amounts of protein were loaded onto 12% sodium dodecyl sulfate-polyacrylamide gel electrophoresis (SDS-PAGE) gels. After electrophoresis, the proteins were efficiently transferred to polyethylene difluoride (PVDF) membranes. These PVDF membranes were blocked by incubating in a solution containing 5% bovine serum albumin (BSA) for 1 hour. After blocking, the membranes were incubated overnight at 4°C with primary antibodies diluted in the blocking solution (5% BSA). Subsequently, the membranes were incubated with secondary antibodies at room temperature for 1 hour. After this incubation, the membranes were imaged using the ECL-enhanced chemoluminescence Kit. Finally, the western blot images were captured with a chemiluminescent imaging system (Sagecreation, Beijing, China). The intensity of each band was quantified by optical densitometry using Image-Pro Plus 6.0 software (Media Cybernetics, USA), allowing for a detailed and quantitative analysis of the protein expression levels.

### Estimation of striatal dopamine levels

Striatal dopamine levels were estimated using a Rat Dopamine ELISA Kit (USCN Life Science Inc., Wuhan, China). All procedures were done according to the manufacturer’s instructions. All samples were diluted (1:2) and added in triplicate to the microtiter plate wells coated with the antibody specific to DA and the plates were incubated for 2h at 37 °C. After washes, a biotin conjugated antibody was added to the wells and the plates incubated for 1h at 37 °C. After washing, wells were incubated with an avidin-conjugated HRP for 30min at 37 °C. Finally, a stop solution was added to stop the reaction, and the color changes were quantified at 450 nm using the BioTek Elisa Reader ELx808. The results were expressed as pg/mg protein.

### Quantitative reverse transcription PCR

Total RNA was isolated from brain tissues using TRIzol (Takara, Japan). The concentration and purity of the extracted RNAs were measured with a Spectrophotometer (Thermo Fisher Scientific, USA). Samples with an absorbance ratio (A260/A280) between 1.8 and 2.0 were deemed appropriate for subsequent experiments. cDNA synthesis was carried out using the SweScript All-in-One RT SuperMix for the qPCR Kit and gDNA Remover Kit, following the manufacturer’s precise protocol. For the amplification of the synthesized cDNA, specific primers were employed in a 10 μl PCR reaction system with the 2×Universal Blue SYBR Green qPCR Master Mix Kit. The primer pairs utilized are presented in **Table 1**. Gene expression analysis was conducted on a QuantStudio™ 6 Flex Realtime-PCR system (Thermo Scientific, USA) using 96-well plates. The reaction conditions included an initial denaturation step at 95°C for 30 s, followed by 40 cycles of denaturation at 95°C for 15 s, annealing/extension at 60°C for 30 s. The total reaction volume was 15 μl. Data analysis was conducted using the 2-ΔΔCt method to calculate the relative ratio of the target gene to the housekeeping gene.

**Table 1:** Primers and their sequences for qRT-PCR.

Primer	Sequence (5'-3')
IL-6 Forward	GAGTTGTGCAATGGCAATTCTG
IL-6 Reverse	ACGGAACTCCAGAAGACCAGAG
TNF-α Forward	TACTGAACTTCGGGGTGATCG
TNF-α Reverse	AGAAGATGATCTGAGTGTGAGGGTC
GAPDH Forward	CTGGAGAAACCTGCCAAGTATG
GAPDH Reverse	GGTGGAAGAATGGGAGTTGCT

### Perfusion and immunohistochemistry

After thirty days post-surgery, the rats were sacrificed and underwent transcardial perfusion with a 4% paraformaldehyde (PFA) solution. Following overnight post-fixation in the same fixative, the brains were gradient dehydrated with ethanol and xylene for cryoprotection. Subsequently, the brains were embedded with paraffin and sectioned at a thickness of 4 µm using a cryostat (Leica, Germany). The substantia nigra pars compacta (SNpc), and striatum sections were cut according to the rat brain atlas by George Paxinos and Charles Watson (sixth edition). These sections were collected and stored at 4°C. For immunohistochemistry, the sections were placed in a 5% hydrogen peroxide (H_2_O_2_) solution in PBS for 15 minutes to quench endogenous peroxidase activity. After being washed, the sections were successively treated with a 0.25% Triton-X 100 solution in PBS, followed by blocking with 5% goat serum for 1.5 hours. Then, they were immunostained with specific primary antibodies overnight at 4°C. After two washes in PBS, the sections were incubated with a peroxidase-conjugated goat anti-rabbit secondary antibody for 2 hours in the dark at room temperature. Finally, the sections were observed under a microscope (Olympus BX51, Japan). After digitalizing the images, counting was manually performed using the Image-Pro Plus 6.0 software.

### Statistical analysis

Statistical analysis was performed using GraphPad Prism 10 software, and data were expressed as the mean ± standard deviation (SD). Differences among all groups were determined using a one-way analysis of variance (ANOVA) followed by a Tukey multiple comparison test. *P* < 0.05 was considered statistically significant.

## Results

### Incretin analogues normalize behavior function in the PD rat model

**Figure 2:**
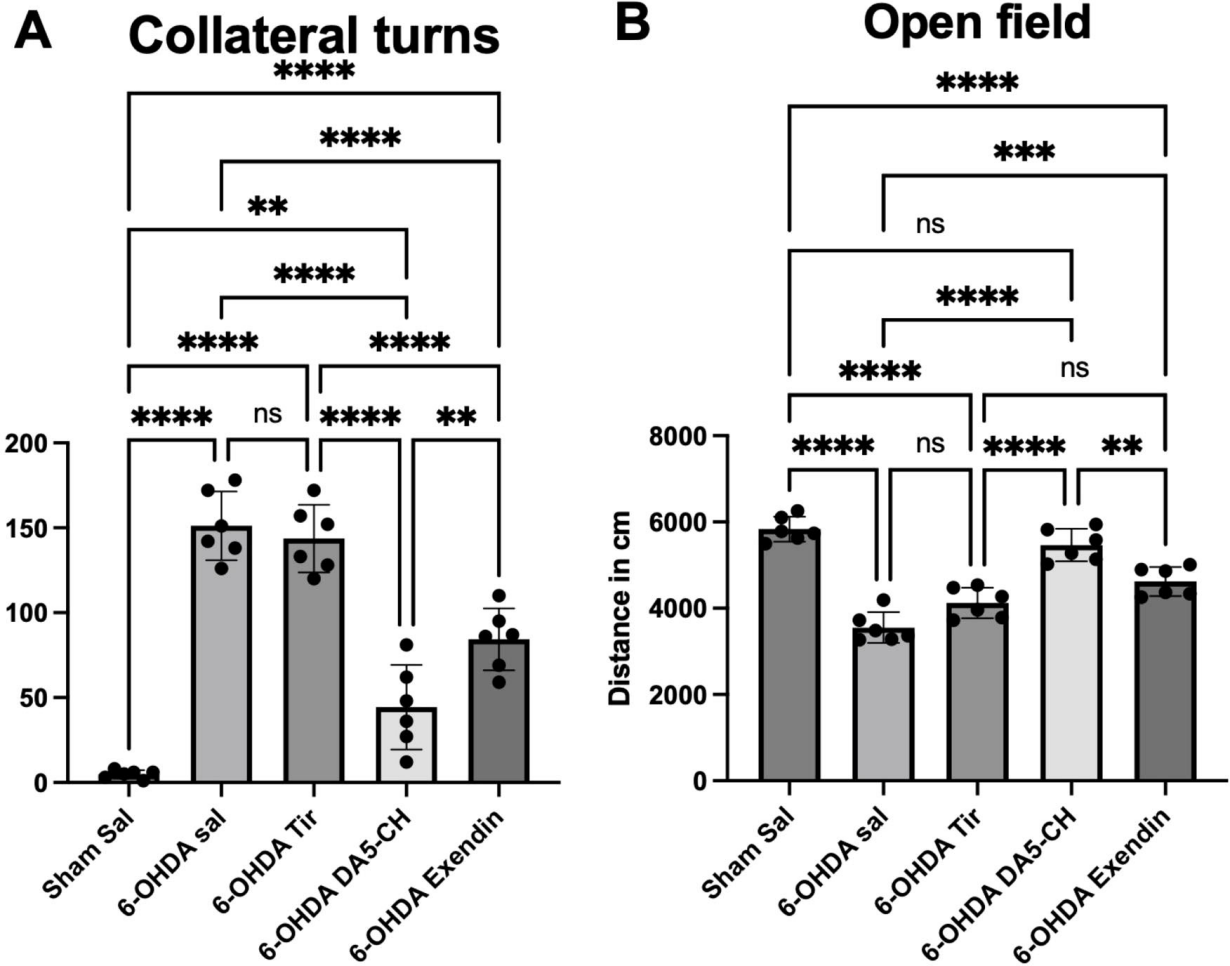
Effects of incretin analogues on motor control in 6-OHDA-induced PD rats. **(A)** Quantitative results of contralateral turns in the apomorphine rotation test. **(B)** Distance run in the open field in 10 min, n=6 per group. **P < 0.01; ***P < 0.001; and ****P < 0.0001(one-way analysis of variance followed by Tukey’s multiple comparison test).

We tested apomorphine-induced rotational behavior to evaluate the functional effects of the lesions and the drug improvements. Ten minutes after the APO injection, the rats rotated with their healthy hind limb (left hind limb). As shown in **Figure 2B**. Compared with the sham-surgery group, the number of rotations in the 6-OHDA model group increased significantly, indicating that the model was successfully established. After drug treatment, the number of rotations decreased significantly, indicating that drug treatment could reduce the number of rotations of 6-OHDA-induced PD rats. Among them, the therapeutic effect of DA5-CH was better than that of Exendin-4, and Exendin-4 was better than that of Tirzepatide (see **Figure 2A**). The one-way ANOVA showed a significant difference (F(4,25)=67.83; p<0.0001) (see **Table 2**).

**Table 2:** Tukey *post-hoc* comparisons.

Sham Sal vs. 6-OHDA sal	p<0.0001
Sham Sal vs. 6-OHDA Tir	p<0.0001
Sham Sal vs. 6-OHDA DA5-CH	p=0.0097
Sham Sal vs. 6-OHDA Exendin	p<0.0001
6-OHDA sal vs. 6-OHDA Tir	p=0.
6-OHDA sal vs. 6-OHDA DA5-CH	p<0.0001
6-OHDA sal vs. 6-OHDA Exendin	p<0.0001
6-OHDA Tir vs. 6-OHDA DA5-CH	p<0.0001
6-OHDA Tir vs. 6-OHDA Exendin	p<0.0001
6-OHDA DA5-CH vs. 6-OHDA Exendin	p<0.0087

### Effects of drug treatment on spontaneous locomotion in the Open Field

General spontaneous activity was measured using a computer tracking system to evaluate if spontaneous activity has gone down after 6-OHDA treatment. The results show that all drugs were able to improve spontaneous activity. DA5-CH was more effective than Exendin-4 and Tirzepatide (see **Figure 2B**; **Table 3**).

**Table 3:** Tukey *post-hoc* comparisons.

Sham Sal vs. 6-OHDA sal	p<0.0001
Sham Sal vs. 6-OHDA Tir	p<0.0001
Sham Sal vs. 6-OHDA DA5-CH	p=0.3584
Sham Sal vs. 6-OHDA Exendin	p<0.0001
6-OHDA sal vs. 6-OHDA Tir	p=0.0551
6-OHDA sal vs. 6-OHDA DA5-CH	p<0.0001
6-OHDA sal vs. 6-OHDA Exendin	p=0.0001
6-OHDA Tir vs. 6-OHDA DA5-CH	p<0.0001
6-OHDA Tir vs. 6-OHDA Exendin	p=0.1202
6-OHDA DA5-CH vs. 6-OHDA Exendin	p=0.0022

The one-way ANOVA showed a significant difference (F(4,25)=44.2; p<0.0001).

Incretin analogues protect dopaminergic neurons in the substantia nigra against 6-OHDA.

Structurally similar to dopamine, 6-OHDA is a neurotoxic compound that can selectively impair dopaminergic neurons, resulting in a substantial loss of these neurons in the substantia nigra (SN). Consequently, it can mimic both the pathological and behavioral features of PD. Tyrosine Hydroxylase (TH) is a crucial enzyme in catalyzing the conversion of tyrosine into L-DOPA (L-3,4-dihydroxy-phenylalanine). Since L-DOPA is a precursor for dopamine synthesis, the expression level of TH directly reflects the functional status of dopaminergic neurons. To assess this dopamine-related injury, we employed immunohistochemistry (IHC) to stain TH in the substantia nigra to quantify the TH expression.

**Figure 3:**
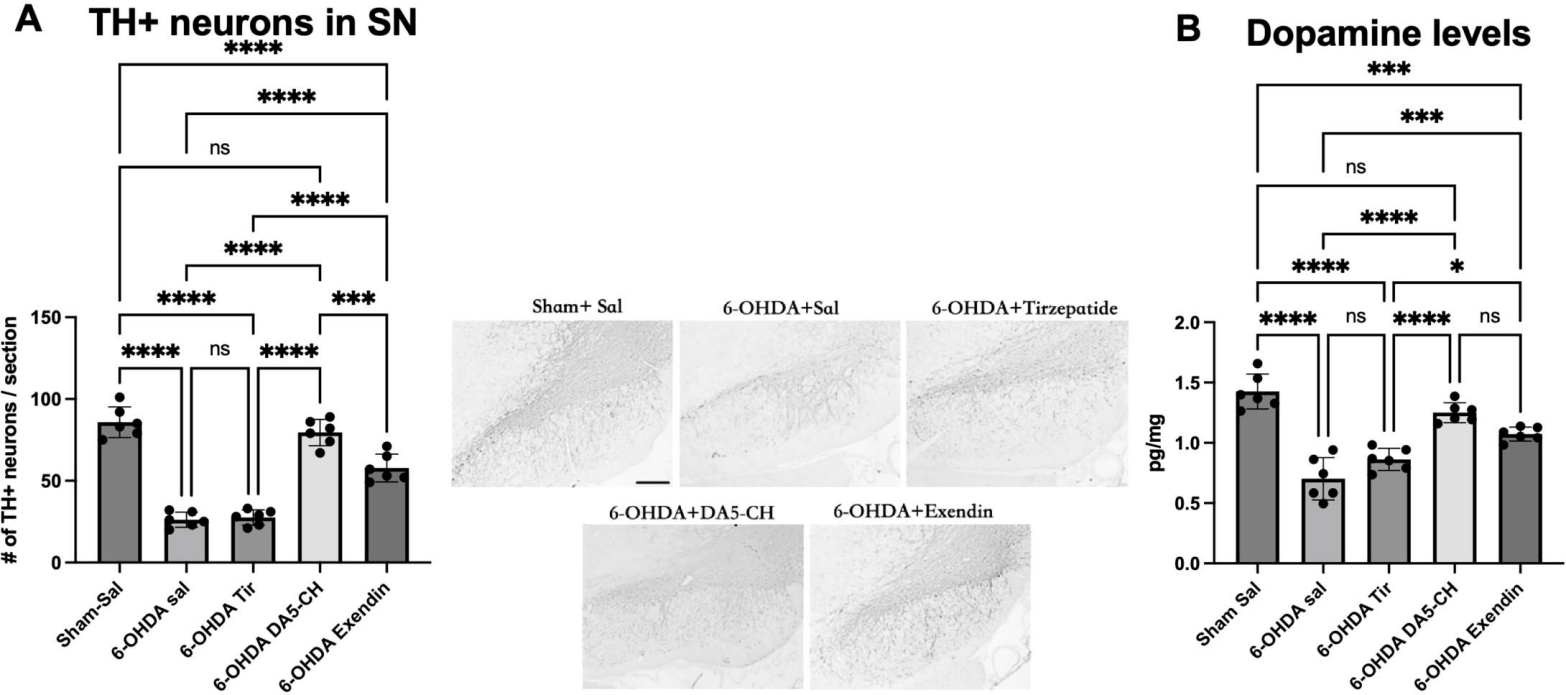
Effects of incretin analogues on TH expression in the SN. **(A)** Quantification of TH positive cells in the SN. Representative IHC images of TH in the SN are shown. Scale bar: 100 μm. **(B)** Dopamine levels in the striatum. Data are represented as the mean ± SD (n = 6). *P < 0.05, ***P < 0.001, and ****P < 0.0001 (one-way analysis of variance followed by Tukey’s multiple comparison test).

As shown in **Figure 3A**, immunohistochemical analysis of TH-positive dopaminergic neurons in the substantia nigra revealed a drastic reduction in cell number in the 6-OHDA-lesioned group compared to the sham-surgery group, indicating severe dopaminergic neurodegeneration. Treatment with DA5-CH significantly mitigated this neuronal loss. Exendin-4 showed some improvements, while Tirzepatide did not show any significant improvement (see **Table 4**).

**Table 4:** Tukey *post-hoc* comparisons.

Sham Sal vs. 6-OHDA sal	p<0.0001
Sham Sal vs. 6-OHDA Tir	p<0.0001
Sham Sal vs. 6-OHDA DA5-CH	p=0.5735
Sham Sal vs. 6-OHDA Exendin	p<0.0001
6-OHDA sal vs. 6-OHDA Tir	p=0.9977
6-OHDA sal vs. 6-OHDA DA5-CH	p<0.0001
6-OHDA sal vs. 6-OHDA Exendin	p<0.0001
6-OHDA Tir vs. 6-OHDA DA5-CH	p<0.0001
6-OHDA Tir vs. 6-OHDA Exendin	p<0.0001
6-OHDA DA5-CH vs. 6-OHDA Exendin	p=0.0002

The one-way ANOVA showed a significant difference (F(4,25)=87; p<0.0001).

### Quantification of dopamine levels in the striatum

To evaluate how much dopamine levels are affected by the reduction of TH expression, we measured dopamine levels in the striatum. 6-OHDA treatment reduced levels significantly, and DA5-CH was able to normalize dopamine levels. DA5-CH was superior to Tirzepatide and Exendin-4 (see **Figure 3B**; **Table 5**).

**Table 5:** Tukey *post-hoc* comparisons.

Sham Sal vs. 6-OHDA sal	p<0.0001
Sham Sal vs. 6-OHDA Tir	p<0.0001
Sham Sal vs. 6-OHDA DA5-CH	p=0.1121
Sham Sal vs. 6-OHDA Exendin	p=0.0002
6-OHDA sal vs. 6-OHDA Tir	p=0.1683
6-OHDA sal vs. 6-OHDA DA5-CH	p<0.0001
6-OHDA sal vs. 6-OHDA Exendin	p=0.0001
6-OHDA Tir vs. 6-OHDA DA5-CH	p<0.0001
6-OHDA Tir vs. 6-OHDA Exendin	p=0.0386
6-OHDA DA5-CH vs. 6-OHDA Exendin	p=0.1018

The one-way ANOVA showed a significant difference (F(4,25)=35; p<0.0001).

### Drug effects on reducing the 6-OHDA-induced chronic inflammation in the brain

Glial fibrillary acidic protein (GFAP) is crucial in constructing the astrocyte cytoskeleton and is exclusively expressed upon astrocyte activation. To assess the activation status of astrocytes, we employed IHC staining of GFAP.

**Figure 4:**
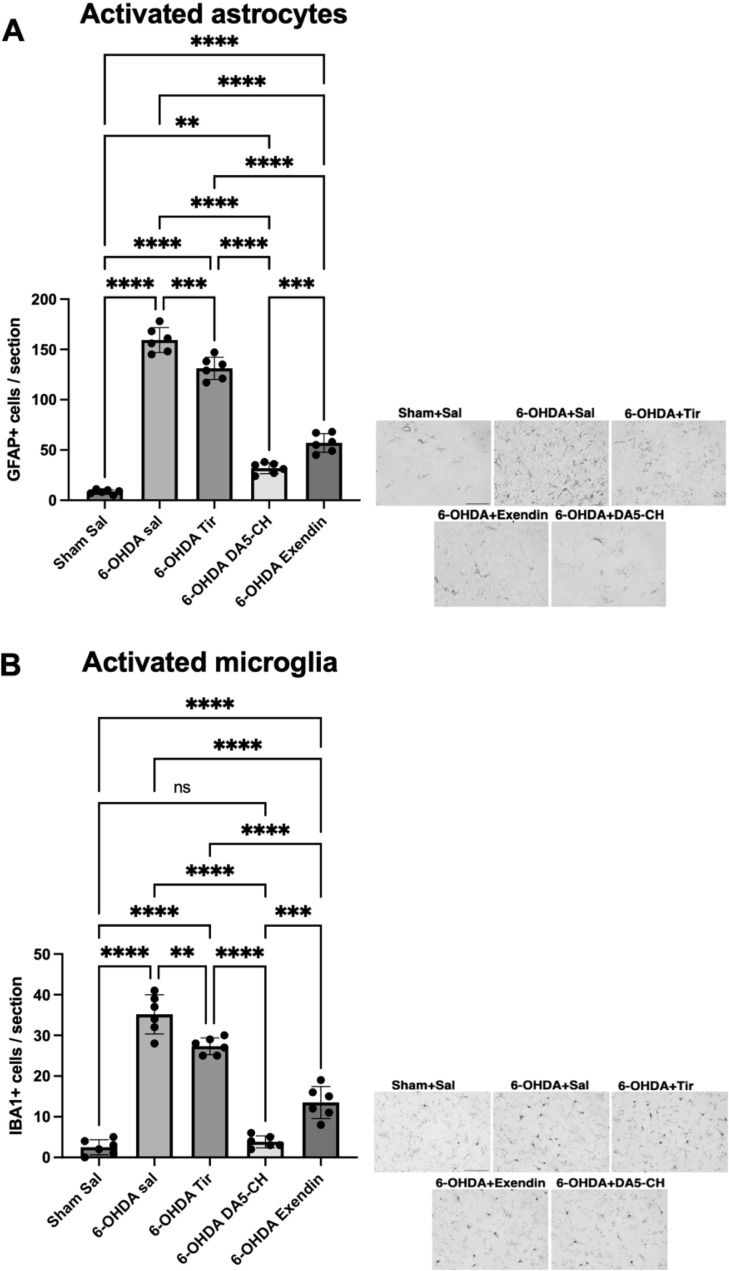
Effects of incretin analogues on 6-OHDA-induced astrocyte and microglia activation in the rat striatum. **(A)** Quantification of GFAP positive cells in the striatum. Representative IHC images of GFAP are shown. Scale bar: 100 μm. **(B)** Quantification of IBA-1 positive cells in the striatum. Representative IHC images are shown. Scale bar: 100 μm. **P < 0.01, ***P < 0.001, and ****P < 0.0001 (one-way analysis of variance followed by Tukey’s multiple comparison test).

As shown in **Figure 4A**, our findings revealed that compared to the sham-surgery group (Sham Saline), the 6-OHDA model group (6-OHDA Saline) exhibited a dramatically elevated number of GFAP(+) astrocytes, indicating robust astrocyte activation induced by 6-OHDA. Following treatment with DA5-CH, there was a significant reduction in GFAP(+) cell count, demonstrating effective inhibition of astrocyte activation. Exendin-4 was more effective than Tirzepatide, but less effective than DA5-CH (see **Table 6**).

**Table 6:** Tukey *post-hoc* comparisons.

Sham Sal vs. 6-OHDA sal	p<0.0001
Sham Sal vs. 6-OHDA Tir	p<0.0001
Sham Sal vs. 6-OHDA DA5-CH	p=0.0010
Sham Sal vs. 6-OHDA Exendin	p<0.0001
6-OHDA sal vs. 6-OHDA Tir	p=0.0001
6-OHDA sal vs. 6-OHDA DA5-CH	p<0.0001
6-OHDA sal vs. 6-OHDA Exendin	p<0.0001
6-OHDA Tir vs. 6-OHDA DA5-CH	p<0.0001
6-OHDA Tir vs. 6-OHDA Exendin	p<0.0001
6-OHDA DA5-CH vs. 6-OHDA Exendin	p=0.0005

The one-way ANOVA showed a significant difference (F(4,25)=31; p<0.0001).

To assess the neuroinflammation triggered by 6-OHDA and the influence of drug treatment on microglial activation, we chose IBA-1, a protein uniquely expressed in microglia that acts as a specific indicator of microglial activation. Subsequently, we measured the expression level IBA-1 in the striatum through IHC staining. As shown in **Figure 4B**, our findings indicate that compared to the sham-surgery group, the 6-OHDA model group exhibited a dramatically elevated number of IBA-1(+) microglia, reflecting robust microglial activation induced by 6-OHDA. DA5-CH was able to reverse or prevent this activation completely. Exendin-4 showed a reduced effect, but was more effective than Tirzepatide (see **Table 7**).

**Table 7:** Tukey *post-hoc* comparisons.

Sham Sal vs. 6-OHDA sal	p<0.0001
Sham Sal vs. 6-OHDA Tir	p<0.0001
Sham Sal vs. 6-OHDA DA5-CH	p=0.9443
Sham Sal vs. 6-OHDA Exendin	p<0.0001
6-OHDA sal vs. 6-OHDA Tir	p=0.0017
6-OHDA sal vs. 6-OHDA DA5-CH	p<0.0001
6-OHDA sal vs. 6-OHDA Exendin	p<0.0001
6-OHDA Tir vs. 6-OHDA DA5-CH	p<0.0001
6-OHDA Tir vs. 6-OHDA Exendin	p<0.0001
6-OHDA DA5-CH vs. 6-OHDA Exendin	p=0.0001

The one-way ANOVA showed a significant difference (F(4,25)=12; p<0.0001).

### Pro-infammatory cytokines in the striatum

**Figure 5:**
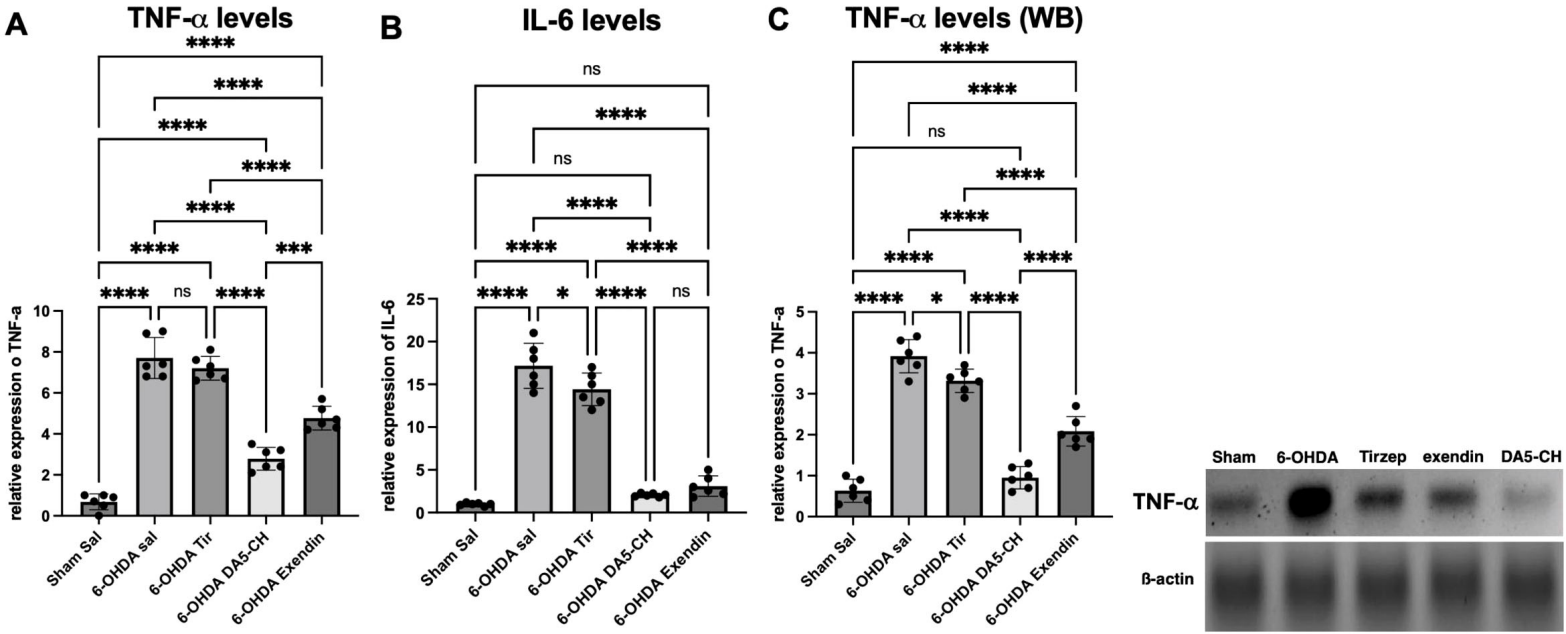
Effects of incretin analogues on 6-OHDA-induced pro-inflammatory cytokine levels in the rat striatum. **(A)** Quantification of TNF-α expression in the striatum using Rt-qPCR. **(B)** Quantification of IL-6 expression in the striatum using Rt-qPCR. **(C)** Quantification of TNF-α expression in the striatum using western blot (n = 4). Sample blots are shown. *P < 0.05, ***P < 0.001 and ****P < 0.0001 (one-way analysis of variance followed by Tukey’s multiple comparison test).

Interleukin (IL) and TNF-α serve as the central drivers of neuroinflammation. They potentially intensify the inflammatory cascade and cause neuronal damage by activating astrocytes and microglia. To assess the impact of cytokines, we employed RT-qPCR and Western blot assay to quantify the expression levels of IL-6 and TNF-α. During the RT-qPCR analysis, as shown in **Figure 5**, our findings revealed a significant upregulation of IL-6 and TNF-α in the 6-OHDA +Sal group compared to the sham-surgery group. However, following treatment with drugs, we observed a notable suppression in the elevated levels of these inflammatory cytokines. Specifically, for IL-6, a highly significant reduction was noted, and for TNF-α, a significant decrease was also evident. At the same time, we obtained similar results in the Western blot assay. As shown in **Figure 5C**, our findings revealed a significant upregulation of TNF-α in the 6-OHDA model group compared to the sham-surgery group (*P* < 0.001). Treatment with DA5-CH was very effective, followed by Exendin-4 that showed some effects also. Tirzepatide was almost ineffective. It showed no effect in the TNF-α PCR quantification and only a small effect in the IL-6 and TNF-α WB evaluation (see **Figure 5**; **Tables 8**–**10**).

**Table 8:** Tukey *post-hoc* comparisons.

Sham Sal vs. 6-OHDA sal	p<0.0001
Sham Sal vs. 6-OHDA Tir	p<0.0001
Sham Sal vs. 6-OHDA DA5-CH	p<0.0001
Sham Sal vs. 6-OHDA Exendin	p<0.0001
6-OHDA sal vs. 6-OHDA Tir	p=0.6776
6-OHDA sal vs. 6-OHDA DA5-CH	p<0.0001
6-OHDA sal vs. 6-OHDA Exendin	p<0.0001
6-OHDA Tir vs. 6-OHDA DA5-CH	p<0.0001
6-OHDA Tir vs. 6-OHDA Exendin	p<0.0001
6-OHDA DA5-CH vs. 6-OHDA Exendin	p=0.0002

**Table 9:** Tukey *post-hoc* comparisons.

Sham Sal vs. 6-OHDA sal	p<0.0001
Sham Sal vs. 6-OHDA Tir	p<0.0001
Sham Sal vs. 6-OHDA DA5-CH	p=0.7544
Sham Sal vs. 6-OHDA Exendin	p=0.1611
6-OHDA sal vs. 6-OHDA Tir	p=0.0381
6-OHDA sal vs. 6-OHDA DA5-CH	p<0.0001
6-OHDA sal vs. 6-OHDA Exendin	p<0.0001
6-OHDA Tir vs. 6-OHDA DA5-CH	p<0.0001
6-OHDA Tir vs. 6-OHDA Exendin	p<0.0001
6-OHDA DA5-CH vs. 6-OHDA Exendin	p=0.7727

**Table 10:** Tukey *post-hoc* comparisons.

Sham Sal vs. 6-OHDA sal	p<0.0001
Sham Sal vs. 6-OHDA Tir	p<0.0001
Sham Sal vs. 6-OHDA DA5-CH	p=0.4615
Sham Sal vs. 6-OHDA Exendin	p<0.0001
6-OHDA sal vs. 6-OHDA Tir	p=0.0285
6-OHDA sal vs. 6-OHDA DA5-CH	p<0.0001
6-OHDA sal vs. 6-OHDA Exendin	p<0.0001
6-OHDA Tir vs. 6-OHDA DA5-CH	p<0.0001
6-OHDA Tir vs. 6-OHDA Exendin	p<0.0001
6-OHDA DA5-CH vs. 6-OHDA Exendin	p<0.0001

TNF-α PCR results: The one-way ANOVA showed a significant difference (F(4,25)=12; p<0.0001).

IL-6 PCR results: The one-way ANOVA showed a significant difference (F(4,25)=14; p<0.0001).

TNF-α WB results: The one-way ANOVA showed a significant difference (F(4,15)=11; p<0.001).

Incretin analogues protect dopaminergic neurons against enhanced 6-OHDA Induced α-Synuclein expression.

**Figure 6:**
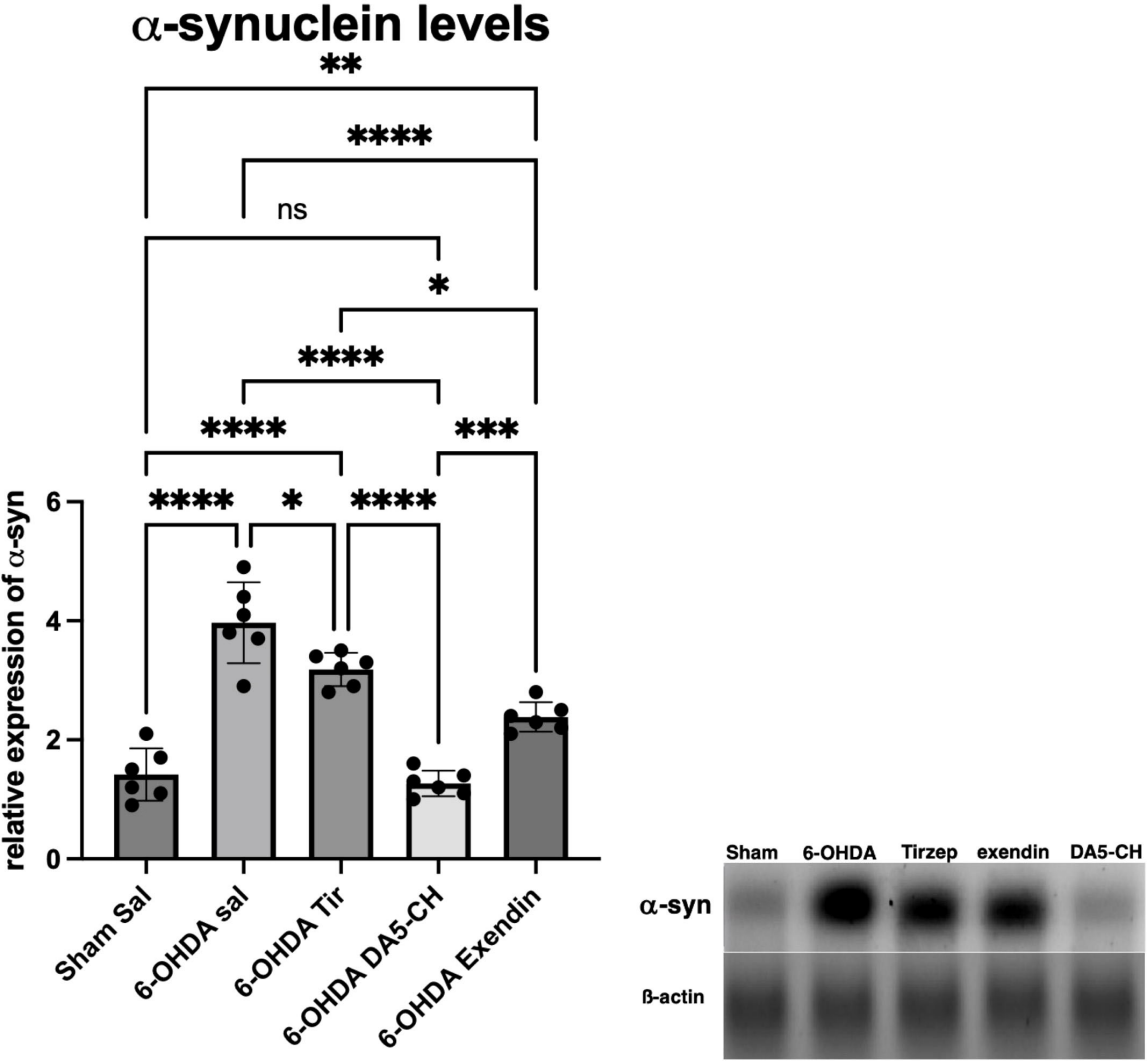
Effects of incretin analogues on α-syn expression in the SN. Quantification of α-syn expression in the SN. Sample western blot bands of α-syn expression in the SN. *P < 0.05, **P < 0.01, ***P < 0.001 and ****P < 0.0001 (one-way analysis of variance followed by Tukey’s multiple comparison test).

To further evaluate α-Synuclein (α-Syn) accumulation, we detected its expression in the substantia nigra through western blot assay. As shown in **Figure 6**, compared with the sham-surgery group, there was a significant increase of α-syn in the substantia nigra of 6-OHDA treated rats. However, DA5-CH treatment reduced α-Syn accumulation back to control levels. Exendin-4 showed a reduced effect compared to DA5-CH, but was more effective than Tirzepate (see **Table 11**).

**Table 11:** Tukey *post-hoc* comparisons.

Sham Sal vs. 6-OHDA sal	p<0.0001
Sham Sal vs. 6-OHDA Tir	p<0.0001
Sham Sal vs. 6-OHDA DA5-CH	p=0.9682
Sham Sal vs. 6-OHDA Exendin	p=0.0034
6-OHDA sal vs. 6-OHDA Tir	p=0.0219
6-OHDA sal vs. 6-OHDA DA5-CH	p<0.0001
6-OHDA sal vs. 6-OHDA Exendin	p<0.0001
6-OHDA Tir vs. 6-OHDA DA5-CH	p<0.0001
6-OHDA Tir vs. 6-OHDA Exendin	p=0.0186
6-OHDA DA5-CH vs. 6-OHDA Exendin	p=0.0007

The one-way ANOVA showed a significant difference (F(4,15)=47; p<0.001).

## Discussion

T2DM has been described as a risk factor for developing PD (4, 23–26). There was an association between insulin resistance and an increased risk of developing PD and dementia, which is a more severe PD phenotype (27). Importantly, insulin desensitization was found in the brains of PD animal models even if they did not have diabetes (1, 28, 29). First clinical data are already available. A phase II clinical trial testing exendin-4 for 48 weeks showed protective effects in PD patients. Motor activity was protected by the drug while the placebo group deteriorated, and the drug effect remained visible 12 weeks after wash-out. DAT-PET brain imaging demonstrated some protection of the dopaminergic nigral-striatal projection, too (5, 6). A phase II trial testing the GLP-1 receptor agonist lixisenatide showed similar effects, with the drug group not deteriorating over 12 months of treatment, while the placebo group deteriorated as expected for this disease. The drug effect was still visible 2 months after the trial had ended (7). In addition, a phase 2 trial testing liraglutide in patients with Alzheimer’s disease reduced cognitive decline (8). We have previously shown in the mouse models of PD that GLP-1 or GIP receptor agonists can protect the brain and normalize motor activity (30–33). Novel dual agonists that can cross the BBB at a better rate than diabetes drugs furthermore showed improved effects over the older drugs (16, 33–36).

The dual agonist tirzepatide has been developed to treat diabetes and to remain in the blood stream for extended periods of time (13, 22). Therefore, these drugs show only limited transfer across the BBB to enter the brain (18, 19, 37, 38). There is a direct correlation between entering the brain and protecting neurons from toxic events by target engagement (18, 19, 35, 39, 40). While exendin-4 can cross the BBB well and show good effects in PD patients, the pegylated version of exendin-4 called NLY01 cannot cross the BBB well (40, 41) and did not show any effects on a phase II trial on PD patients in the primary and secondary readouts (42). The addition of the 40kDa pegylation prevented the crossing of the BBB and therefore did not have any effects.

We therefore developed dual GLP-1/GIP receptor agonists that can cross the BBB at an enhanced rate (15, 33, 40). Our dual agonist DA5-CH has shown superior neuroprotective effects to exendin-4 in the 6-OHDA rat model of PD (43) and to semaglutide in the same rat model (21).

The 6-OHDA unilateral lesion MBF rat model develops PD-like motor deficits and shows loss of dopaminergic neurons in the substantia nigra (34, 43, 44). Thus, in order to evaluate the neuroprotective potential of Tirzepatide, we wanted to test this drug in the 6-OHDA lesion PD rat model. We tested both drugs head-to-had at equimolar doses to investigate which drug is more effective at a DA5-CH dose that previously had shown good effects in PD animal models (16). Exendin-4 was tested alongside as a comparator.

In the present study, exendin-4 and DA5-CH reduced the circling behavior when apomorphine was given to activate dopamine receptors, which showed that the lesioned hemisphere was functional again after drug treatment. Exendin-4 was less effective than DA5-CH. Tirzepatide did not show any effect, demonstrating that this dual GLP-1/GIP receptor agonist did not get into the brain at doses required for target engagement. In contrast, the dual GLP-1/GIP receptor agonist DA5-CH that can cross the BBB well was very effective. The same pattern was seen in the Open Field spontaneous locomotion test. When quantifying dopaminergic neurons in the substantia nigra, DA5-CH showed superior neuroprotective effects than exendin-4 or tirzepatide. When measuring dopamine levels in the striatum, DA5-CH again showed better effects than Exendin-4, and Tirzepatide was ineffective. This is again most likely due to the fact that DA5-CH can cross the BBB more easily than tirzepatide and can activate the receptors in the brain to induce the neuroprotective effect. Exendin-4 showed some protective effect as it can cross the BBB fairly well, but it is only a GLP-1 receptor agonist.

Chronic inflammation in the brain is a key driver in PD disease progression (45, 46). Activated microglial release proinflammatory cytokines that can damage synaptic activity, increase oxidative stress and reduce glucose uptake and energy utilization in neurons (47–52). The reduction of the inflammatory response by these drugs is due to the fact that glia cells express the GLP-1 receptor, and that GLP-1 acts as an anti-inflammatory cytokine (53, 54). The reduction of TNF-α and IL-1ß levels in the brain will contribute to the reversal of insulin de-sensitization that we previously observed in the 6-OHDA lesioned rats, where TNF-α reduced IRS-1 serine phosphorylation and thus inhibits its function (21, 55). Previous studies also have shown insulin re-sensitization with GLP-1 receptor agonists (56–59). In our study, DA5-CH reduced the inflammation more effectively than Exendin-4. Tirzepatide was less effective.

It has been suggested that a key feature in the pathology of PD is the aggregation of α-synuclein, a component of Lewy bodies in FTD patients (60). In our study, a reduction of the α-synuclein levels was observed after drug treatment, with DA5-CH being the most effective. We have previously shown that in the MPTP mouse model, α-synuclein expression is very much increased, and that DA5-CH (40) can reduce the levels in the brain. We had furthermore shown that DA5-CH can reduce α-synuclein levels in the brain of 6-OHDA treated rats better than semaglutide (21).

While DA5-CH showed better effects in reducing the inflammation response, it is not surprising that Tirzepatide did not show comparable effects in these parameters. In a previous study in a mouse model of Alzheimer’s disease, Tirzepatide did not show any protective effects and did not reduce inflammation in the brain either (61). In the rotenone rat model of PD, Tirzepatide did show neuroprotective effects, but the doses used were much higher (50 or 100nmol/kg s.c. once-daily) and do not correspond to the much lower doses used in the clinic (62). Importantly, DA5-CH showed superior effects compared to Exendin-4 in our study, which already has shown protective effects in PD patients (5), suggesting that DA5-CH would be more effective in treating PD patients.

In conclusion, the dual GLP-1/GIP receptor agonist DA5-CH that can cross the BBB at an enhanced level is superior to the dual agonist Tirzepatide that cannot cross the BBB readily and the single GLP-1R agonist exendin-4 that can cross the BBB well but only activates the GLP-1 receptor. We therefore postulate that DA5-CH would have superior neuroprotective effects in treating Parkinson’s disease compared to the other drugs.

## Data Availability

The original contributions presented in the study are included in the article/supplementary material. Further inquiries can be directed to the corresponding author.
